# Outcome measures reported in abstracts of randomized controlled trials in leading clinical journals: A bibliometric study

**DOI:** 10.1002/jgf2.306

**Published:** 2020-04-30

**Authors:** Takeshi Seta, Yoshimitsu Takahashi, Yukitaka Yamashita, Masahiro Hiraoka, Takeo Nakayama

**Affiliations:** ^1^ Department of Gastroenterology and Hepatology Japanese Red Cross Wakayama Medical Center Wakayama Japan; ^2^ Department of Health Informatics Kyoto University School of Public Health Kyoto Japan; ^3^ President of Japanese Red Cross Wakayama Medical Center Wakayama Japan

**Keywords:** absolute risk, abstract, effect measurement, relative risk, submission guidelines

## Abstract

**Backgrounds:**

The CONSORT for Abstracts checklist published in 2008 recommends that authors report effect size for their studies. Meanwhile, the FDA strongly recommends reporting both ratio and difference measures. However, the measures of effect used in recent clinical trial reports remain unknown. This study is aimed to reveal trends regarding the measures of effect of interventions described in abstracts of recent randomized controlled trials (RCTs) in leading journals.

**Methods:**

A bibliometric analysis of data was obtained by electronic searches. Human RCTs published in 2016 in the following five journals were searched using PubMed: Annals of Internal Medicine, British Medical Journal, Journal of American Medical Association, The Lancet, and New England Journal of Medicine. Main outcome is numbers of studies reporting each measure in their abstracts.

**Results:**

Among abstracts of 334 articles, measures most frequently used were relative risk alone (n = 169), followed by absolute risk alone (n = 92), and raw data alone (n = 58). Reporting of the following measures was relatively limited: both ratio and difference measures (n = 8), raw data with ratio measures (n = 5), and raw data with difference measures (n = 2). None of the studies reported raw data with both ratio and difference measures. Only 15 articles described multiple measures of effect in their abstracts.

**Conclusions:**

More than half of the RCT abstracts published in the five leading journals in 2016 reported risk ratio alone to indicate effect size. Even abstracts in the five leading journals did not adhere fully to the CONSORT for Abstracts or FDA recommendations.

## Graphical abstract

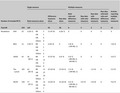

## 1 INTRODUCTION

Risk ratio (RR), or relative risk, means the ratio of two risks, usually of exposed and unexposed.^1^ Risk difference (RD), or attributable risk, means the difference of two risks in the exposed and in the unexposed. When the probability of primary endpoint of both groups is low, for example, in the issue of primary prevention, RR looks larger than RD. The ratio index is concerned to exaggerate the effect size compared with the absolute value or difference index.^2^, ^3^ The absolute value is inevitable for evaluating the size of the problem. Despite repeated recommendation to report both the RR and the RD,^4^, ^5^, ^6^, ^7^, ^8^ RD has been relatively underreported.^9^, ^10^, ^11^

Abstracts of medical articles must contain accurate descriptions of contents with sufficient transparency, as many readers use abstracts to obtain efficiently the necessary information. The CONSORT statement for abstracts published in 2008 lists items that authors should consider when writing an abstract to report results of their randomized controlled trials (RCTs) in medical journals.^12^, ^13^ In this statement, the use of estimated effect sizes (ie, relative risk [RR], relative risk reduction [RRR], odds ratio [OR], hazard ratio [HR], and risk difference [RD]) is recommended to describe study outcomes. For example, PLOS Medicine suggests reporting raw data and RR along with 95% confidence intervals.^13^ The FDA has repeatedly noted that ratio measures tend to emphasize effects more than difference do, even when the same data are used, and that interpreting results requires caution.^2^, ^9^, ^10^ It remains unknown as to how effect size is reported in abstracts of recent RCTs conducted in various fields. In this study, we reviewed the submission guidelines of representative clinical medicine journals regarding the abstract for RCTs and aimed to clarify the measures of effect reported in the abstracts published in these journals.

## 2 MATERIALS AND METHODS

### 2.1 Target journals

We checked the impact factor in Journal of Citation Reports of Web of Science with the category of "MEDICINE, GENERAL & INTERNAL", in 2016.

The top five journals were selected by referring to the impact factor as target journals. Thus, in this study, human RCTs published in January through December 2016 in the Annals of Internal Medicine (AIM), British Medical Journal (BMJ), Journal of American Medical Association (JAMA), The Lancet (hereafter, Lancet), and New England Journal of Medicine (NEJM) were selected.

### 2.2 Extraction method

The final literature search was conducted in June 2018. Database searches were performed using PubMed, with the following search formula: “Randomized Controlled Trial” [Publication Type] Filters: Publication date from 2016/01/01 to 2016/12/31; Humans. Each journal name was used as a keyword in the above search formula, and the RCT of each journal was searched.

### 2.3 Inclusion and exclusion criteria

Among articles retrieved electronically, abstracts that did not clarify the type of study (ie, RCT or not) and those reporting RCTs that were not related to treatment or prevention (eg, diagnosis) were excluded. If clinical effects were assessed using multiple measures within one study, only the primary endpoint was subjected to evaluation. If the primary endpoint was assessed using multiple effect measures, only the first effect measure mentioned was subjected to evaluation.

### 2.4 Classification of intervention purpose

Randomized controlled trials ultimately included in this study were classified according to the purpose of interventions (treatment or prevention) based on the primary outcome described in the methods section of each article.

### 2.5 Primary outcome

We read through the abstracts of all included studies and counted the numbers of articles that described the primary endpoint in terms of ratio measures (eg, RR, HR, OR), difference measures (RD), raw data alone, both ratio and difference measures, raw data with ratio measures, raw data with difference measures, and raw data with both ratio and difference measures. In addition, the number of characters in the abstract of the final inclusion RCTs published on PubMed was counted.

### 2.6 Evaluation of submission guidelines

We checked submission guidelines within journals (from 2014 through 2016) or on the web site (as of June 2018) of each journal. When these approaches were unsuccessful, email inquiries were sent to the editorial office of each journal via the “Contact us” link on the web site. If no responses were received, a reminder was sent two weeks after the first inquiry; no further contact was made after this, regardless of the absence of any response.

### 2.7 Author roles

The first author (TS) designed the study and prepared the study protocol through discussions with YT. TS extracted data necessary for the analysis from the target journals (this included determining whether the included RCTs were treatment‐ or prevention‐focused studies). Following this, TN checked all the processes performed by TS and asked questions, if any; differences in opinion were resolved through discussion. YY and MH read and proofread the finished manuscript. All authors approved the final version of the submitted manuscript.

### 2.8 Statistical analysis

We evaluated how many times each measure of effect was used to describe the primary endpoint in the abstracts of the included RCTs and assessed differences among journals using the chi‐squared test. The Bonferroni correction for multiple comparisons was used to test differences among the five journals regarding 10 items. A *P *< .005 was considered statistically significant. All statistical analyses were performed using Stata ver. 14.

## 3 RESULTS

### 3.1 Electronic searches

**Figure 1:**
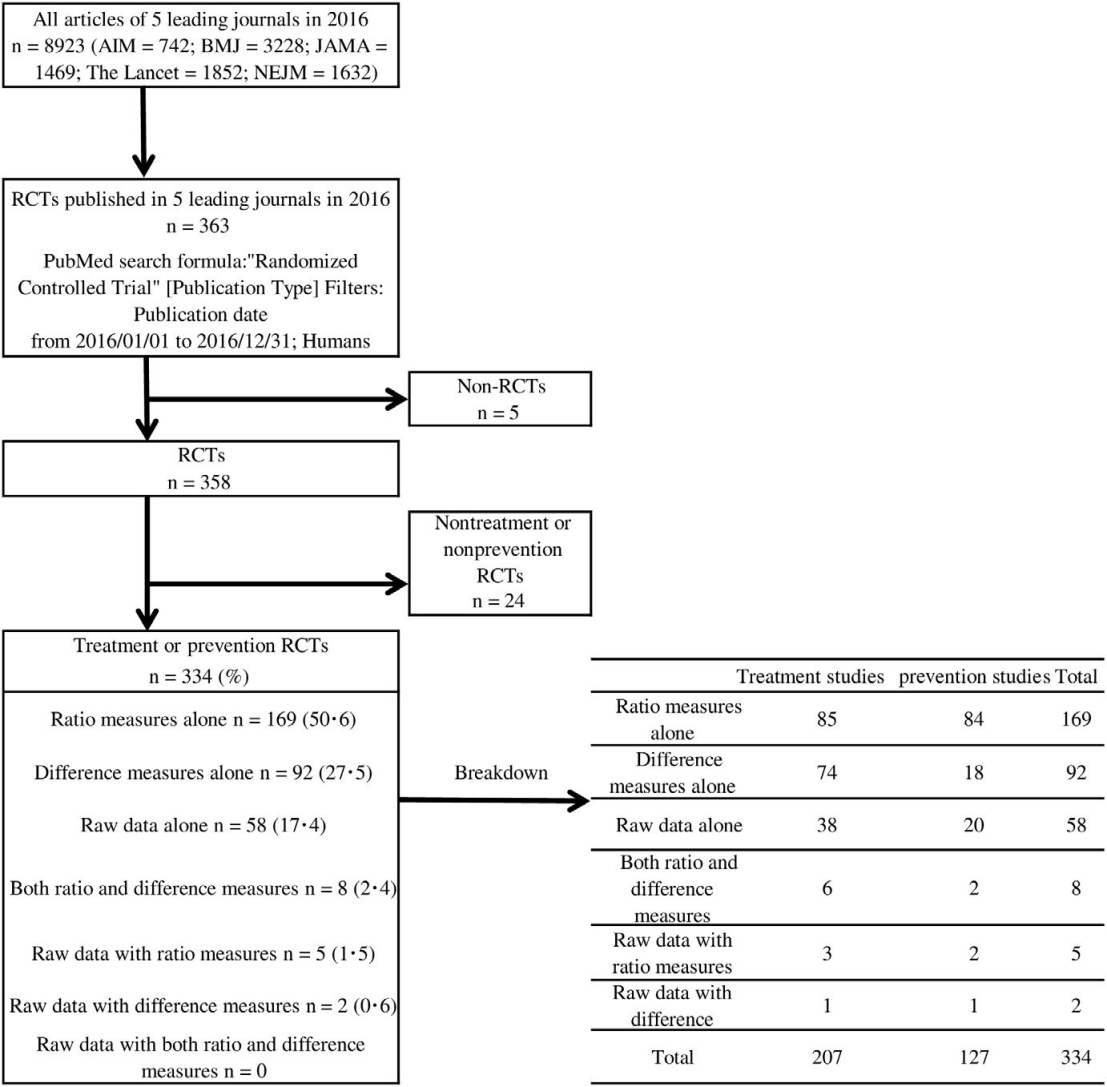
Flow of selection leading to the final article

Among 363 RCT articles extracted from five leading journals in 2016, we read through the abstracts of 334 articles identified to meet the inclusion criteria, excluding five non‐RCT reports (which could not be determined from the abstracts) and 24 RCT articles with objectives other than prevention/treatment (determined from the abstracts; Figure 1). In addition to RR, HR, and OR, attack rate ratio, incidence rate ratio, recurrence ratio, and rate ratio were used as ratio measures; these were all accepted as ratio measures in this study. We identified 169 abstracts reporting relative risk alone, 92 reporting absolute risk alone, 58 reporting raw data alone, eight reporting both ratio and difference measures, five reporting raw data with ratio measures, and two reporting raw data with difference measures. None of the journals had abstracts reporting raw data with both ratio and difference measures.

### 3.2 Measures of effect used in the abstracts of RCTs subjected to analysis

Table 1 summarizes the results by journal as follows: AIM, 23; BMJ, 12; JAMA, 64; Lancet, 105; and NEJM, 130. The proportion of abstracts reporting ratio measures alone was highest for Lancet, accounting for 60･0% of all RCTs, followed by NEJM (59･2%), BMJ (41･7%), JAMA (28･1%), and AIM (26･1%). The proportion of abstracts reporting difference measures alone was highest for JAMA (57･8%), followed by BMJ (50･0%), AIM (47･8%), Lancet (23･8%), and NEJM (10･0%). The proportion of abstracts reporting raw data alone was highest for AIM (21･6%), followed by NEJM (23･8%), Lancet (14･2%), JAMA (9･4%), and BMJ (0%). Overall, 15 (4･5%) abstracts reported multiple measures of effect as recommended by the CONSORT for Abstracts; the highest number of abstracts was found in NEJM (9), followed by JAMA (3), Lancet (2), BMJ (1), and AIM (0).

**Table 1:** Details of studies presenting ratio measures, difference measures, and raw data to describe effect size (Overall)

Number of included RCTs			Single measure					Multiple measures				Articles reporting multiple effect measures	Trend of each journal on the number of articles reporting multiple effect measures (p value)					Submission guidelines			Analyses of Abstracts			
			Ratio measures alone			Difference measures alone	Raw data alone	Both ratio and difference measures	Raw data with ratio measures	Raw data with difference measures	Raw data with both ratio and difference measures							Requirement to report absolute risk or calculate NNT		Word limit for abstracts	Number of papers within the word limit for abstracts	Median of number of words in abstract	Median	
Total ##		334	169			92	58	8	5	2	0	15	AIM	BMJ	JAMA	The Lancet	NEJM	2014‐2016	Present				Lower	Upper
Breakdown	AIM	23	6 (26･1)	RR	5	11 (47･8)	6 (26･1)	0	0	0	0	0	‐	0･343	0･563	1･000	0･36	Only the current URL address was provided	Yes	275 words max.	9 (39.1)	291	228	336
				HR	1																			
				OR	0																			
				Others	0																			
	BMJ	12	5 (41･7)	RR	1	6 (50･0)	0	1 (8･3) （HR+RD, 1)	0	0	0	1 (8･3)	0･343	‐	0･51	0･279	0･6	Guidelines from 2014‐2016 no longer exist	Yes	250‐300 words^a^	5 (41.7)	370	279	427
				HR	1																			
				OR	1																			
				Others	2																			
	JAMA	64	18 (28･1)	RR	4	37 (57･8)	6 (9･4)	3 (4･7) （RR+RD, 2; HR+RD 1)	0	0	0	3 (4･7)	0･563	0･51	‐	0･368	0･75	Instructs authors to report quantified results to the extent possible	Yes	350 words max.	8 (12.5)	410	321	526
				HR	11																			
				OR	3																			
				Others	0																			
	The Lancet	105	63 (60･0)	RR	11	25 (23･8)	15 (14･2)	1 (1･0) （HR+RD, 1)	0	1 (1･0)	0	2 (1･9)	1･000	0･279	0･37	‐	0･12	Only information currently available exists	Yes	300 words max.	4 (3.8)	442	269	689
				HR	37																			
				OR	10																			
				Others	5																			
	NEJM	130	77 (59･2)	RR	12	13 (10･0)	31 (23･8)	3 (2･3) （HR+RD, 2; RR+RD 1)	5 (3･9)	1 (0･8)	0	9 (6･9)	0･36	0･6	0･75	0･117	‐	Only the current URL address was provided	No specification	250 words max.	5 (3.8)	327	224	490
				HR	54																			
				OR	7																			
				Others	4																			

Note

Numbers in ( ) show percentage of RCTs, with the number of all included RCTs by journal as the denominator.

Abbreviations: AIM, Annals of Internal Medicine; BMJ, British Medical Journal; HR, hazard ratio; JAMA, Journal of American Medical Association; NEJM, New England Journal of Medicine; OR, odds ratio; RCT, randomized controlled trial; RR, relative risk (risk ratio); RD, risk difference.

^a^ The guidelines note the following: "Abstracts should be 250‐300 words long: you may need up to 400 words, however, for a CONSORT or PRISMA style abstract."

### 3.3 Differences between treatment studies and prevention studies

As shown in Table 2, among 207 articles on treatment studies, 85 reported relative risk alone, 74 reported absolute risk alone, 38 reported raw data alone, six reported both ratio and difference measures, three reported raw data with ratio measures, and one reported raw data with difference measures. The proportions of articles reporting ratio measures alone, difference measures alone, and raw data alone were highest for Lancet (32/62; 51･6%), JAMA (30/45; 66･7%), and NEJM (20/71; 28･2%), respectively. Overall, 10 articles reported multiple measures, of which six were published in NEJM.

**Table 2:** Details of studies presenting ratio measures, difference measures, and raw data to describe effect size (By study type)

Overall n = 334																			
Journal name	Number of included RCTs	Treatment studies n = 207								Journal name	Number of included RCTs	Prevention studies n = 127							
		Single measure					Multiple measures					Single measure					Multiple measures		
		Ratio measures alone			Difference measures alone	Raw data alone	Both ratio and difference measures	Raw data with ratio measures	Raw data with difference measures			Ratio measures alone			Difference measures alone	Raw data alone	Both ratio and difference measures	Raw data with ratio measures	Raw data with difference measures
AIM	19	5 (26･3)	RR	5	9 (47･4)	5 (26･3)	0	0	0	AIM	4	1 (25･0)	RR	0	2 (50･0)	1 (25･0)	0	0	0
			HR	0									HR	1					
			OR	0									OR	0					
			Others	0									Others	0					
BMJ	10	3 (30･0)	RR	1	6 (60･0)	0	1 (10･0) (HR+RD, 1)	0	0	BMJ	2	2 (100)	RR	0	0	0	0	0	0
			HR	0									HR	1					
			OR	1									OR	0					
			Others	1^a^									Others	1^b^					
JAMA	45	9 (20･0)	RR	2	30 (66･7)	4 (8･9)	2 (4･4) (RR+RD, 2)	0	0	JAMA	19	9 (47･4)	RR	2	7 (36･8)	2 (10･5)	1 (5･3) (HR+RD, 1)	0	0
			HR	5									HR	6					
			OR	2									OR	1					
			Others	0									Others	0					
The Lancet	62	32 (51･6)	RR	4	20 (32･3)	9 (14･5)	1 (1･6) (HR+RD, 1)	0	0	The Lancet	43	31 (72･1)	RR	7	5 (11･6)	6 (14･0)	0	0	1 (2･3)
			HR	23									HR	14					
			OR	4									OR	6					
			Others	1^c^									Others	4^d^					
NEJM	71	36 (50･7)	RR	3	9 (12･7)	20 (28･2)	2 (2･8) (HR+RD, 2)	3 (4･2)	1 (1･4)	NEJM	59	41 (69･5)	RR	9	4 (6･8)	11 (18･6)	1 (1･7) (RR+RD, 1)	2 (3･4)	0
			HR	28									HR	26					
			OR	3									OR	4					
			Others	2^e^									Others	2^f^					

Note

Numbers in parentheses indicate the percentage of RCTs, with the number of all included RCTs by journal as the denominator.

Abbreviations: AIM, Annals of Internal Medicine; BMJ, British Medical Journal; HR, hazard ratio; JAMA, Journal of American Medical Association; NEJM, New England Journal of Medicine; OR, odds ratio; RCT, randomized controlled trial; RD, risk difference; RR, relative risk (risk ratio).

^a^ Attack rate ratio.

^b^ Incidence rate ratio.

^c^ Recurrence ratio.

^d^ 2 rate ratio; 2 incidence rate ratio.

^e^ 2 rate ratio.

^f^ 2 rate ratio

Among 127 articles on prevention studies, 84 reported relative risk alone, 18 reported absolute risk alone, 20 reported raw data alone, two reported both ratio and difference measures, two reported raw data with ratio measures, and one reported raw data with difference measures. The proportions of articles reporting ratio measures alone, difference measures alone, and raw data alone were highest for BMJ (2/2; 100%), AIM (2/4; 50%), and AIM (1/4; 25%), respectively. Overall, five articles reported multiple measures, of which three were published in NEJM.

The proportion of prevention studies reporting absolute risk was significantly lower than that of treatment studies (prevention studies: 18/127, 14･2% vs treatment studies: 74/207; 35･7%; *P *< .01).

### 3.4 Statistical test for rates of use of multiple effect measures among journals

The results of the chi‐squared test revealed no significant differences in the rates of use of multiple effect measures among journals (Table 1).

### 3.5 Number of characters in the abstract of the final inclusion RCTs

Abstracts of all 334 RCTs used in this study were obtained from PubMed, and the number of characters in the abstract was counted. As a result, not only BMJ but also other four journals exceeded the upper limit of the number of characters in abstracts (Table 1).

### 3.6 Evaluation of submission guidelines

Only JAMA included submission guidelines in the journal publication itself (eg, the December 2016 issue; Table 1). As of June 2018, all journals were confirmed to provide an electronic version of submission guidelines on the respective web sites, although guidelines pertaining to 2014‐2016 publications could not be accessed. Accordingly, we contacted the editorial offices of four journals (AIM, BMJ, Lancet, and NEJM) via email and obtained responses from all journals. BMJ had no materials that could be provided to us, and Lancet only had current submission guidelines available (as of June 2018). Both AIM and NEJM provided URL addresses that led us to their current submission guidelines (June 2018), with no clear details. Only JAMA submission guidelines from 2014‐2016 stipulated that “absolute values or quantified results should be shown.” The other four journals had unclear or no descriptions. The word limit for abstracts was 275 for AIM, 250‐300 for BMJ, 350 for JAMA, 300 for Lancet, and 250 for NEJM. As for BMJ, the following comment was also included in the main text of its guidelines: “Abstracts should be 250‐300 words long: you may need up to 400 words, however, for a CONSORT or PRISMA style abstract.”

## 4 DISCUSSION

These findings suggest that the recommendations of the CONSORT for Abstracts, FDA to present results along with effect measures, or absolute numbers had not fully permeated in 2016 when those articles were published.

Why is the “ratio measurement” preferred? First, the reason the “ratio” is more popular than the “difference” is the large amount of information indicated by the “ratio” in the comparison of the results of the two groups. For example, the “difference between the two groups” is 5%. The difference between “1% and 6%” is 5%, and “90% and 95%” is 5%. If the former is indicated by a ratio, it is 6 times, and the latter about 1.05. Comparing the difference and the ratio, it is estimated that the ratio is more likely to give the reader an image of the research results. In the comparison of two groups, it seems that the ratio is preferred when selecting one of the difference or the ratio.

Second, this is not just a matter of awareness of individual researchers: Among the five journals examined in the present study, NEJM and Lancet tended to prefer ratio, whereas AIM, BMJ, and JAMA tended to prefer difference. Furthermore, if the word limit for abstracts is low, contents that can be described also become limited. As for BMJ, the guidelines specifically state that CONSORT‐style abstracts may be up to 400 words in length.^14^ Thus, the results obtained in this study might have been greatly affected by the submission guidelines of each journal.

At the time of submission, the number of characters is within the regulation, and at the time of publication, the reason for exceeding the number of characters may be the reflection of comments at the time of peer review. The submission guidelines are only instructions at the time of admission, and at the time of publication, the number of characters (respecting the comments of the reviewers) is decided by editors. The number of characters of abstract of a published article exceeds the specified number of characters (perhaps in the process of responding to peer review) from our result. Reviewers who do not dictate the addition of additional indicators for differences or absolute values are likely to be responsible.

There are several limitations in this study. First, we could not identify submission guidelines from 2016 for four of the five leading journals (excluding JAMA) when we checked as of June 2018. Thus, the details of the submission instructions to which the authors may have referred in preparing their abstracts (ie, 2016) are unclear. Second, as the present study only included abstracts published in selected journals over the course of one year, the results may not be representative of all medical articles. Therefore, generalizing the results needs care.

It is desirable that each journal provide submission instructions for authors to report their results “along with multiple effect measures,” as this would promote the dissemination of and adherence to the recommendations of the CONSORT for Abstracts and FDA.

## CONFLICT OF INTERESTS

The authors have stated explicitly that there are no conflicts of interest in connection with this article.

## References

[1] Porta M, editor. A Dictionary of Epidemiology, 6th edn Oxford: Oxford University Press, 2014.

[2] Fagerlin A . Chapter 7: Quantitative information In: Fischhoff B, Brewer NT, Downs JS, editors. Communicating Risks and Benefits: An Evidence‐Based User's Guide. Annapolis, MD: Food and Drug Administration (FDA), 2011; p. 57–61. Available from http://www.fda.gov/downloads/AboutFDA/ReportsManualsForms/Reports/UCM268069.pdf

[3] Naylor CD , Chen E , Strauss B . Measured enthusiasm: does the method of reporting trial results alter perceptions of therapeutic effectiveness? Ann Intern Med. 1992;117:916–21. 1443954 10.7326/0003-4819-117-11-916

[4] Elting LS , Martin CG , Cantor SB , Rubenstein EB . Influence of data display formats on physician investigators' decisions to stop clinical trials: prospective trial with repeated measures. BMJ. 1999;318:1527–31. 10356010 10.1136/bmj.318.7197.1527 PMC27896

[5] Schulz KF , Altman DG , Moher D , CONSORT Group . CONSORT statement: updated guidelines for reporting parallel group randomized trials. Ann Intern Med. 2010;2010(152):726–32. 10.7326/0003-4819-152-11-201006010-00232 20335313

[6] Nuovo J , Melnikow J , Chang D . Reporting number needed to treat and absolute risk reduction in randomized controlled trials. JAMA. 2002;287:2813–4. 12038920 10.1001/jama.287.21.2813

[7] Schechtman E . Odds ratio, relative risk, absolute risk reduction, and the number needed to treat–which of these should we use? Value Health. 2002;5:431–6. 12201860 10.1046/J.1524-4733.2002.55150.x

[8] Citrome L . Relative vs. absolute measures of benefit and risk: what's the difference? Acta Psychiatr Scand. 2010;121:94–102. 19694632 10.1111/j.1600-0447.2009.01449.x

[9] Nakayama T . Under‐reporting of attributable risk and reporting of the risk ratio in epidemiologic literature. Epidemiology. 2000;11:366–7. 10784264 10.1097/00001648-200005000-00032

[10] Nakayama T , Zaman MM , Tanaka H . Reporting of attributable and relative risks, 1966–97. Lancet. 1998;351:1179. 9643696 10.1016/S0140-6736(05)79123-6

[11] Gigerenzer G , Wegwarth O , Feufel M . Misleading communication of risk. BMJ. 2010;341:c4830. 20940219 10.1136/bmj.c4830

[12] Hopewell S , Clarke M , Moher D , Wager E , Middleton P , Altman DG , et al. CONSORT Group. CONSORT for reporting randomised trials in journal and conference abstracts. Lancet. 2008;371:281–3. 18221781 10.1016/S0140-6736(07)61835-2

[13] Hopewell S , Clarke M , Moher D , Wager E , Middleton P , Altman DG , et al. CONSORT Group. CONSORT for reporting randomized controlled trials in journal and conference abstracts: explanation and elaboration. PLoS Med. 2008;5:e20. 18215107 10.1371/journal.pmed.0050020 PMC2211558

[14] Available from https://www.bmj.com/about‐bmj/resources‐authors/article‐types

